# Melanopsin phototransduction: beyond canonical cascades

**DOI:** 10.1242/jeb.226522

**Published:** 2021-11-29

**Authors:** Ely Contreras, Alexis P. Nobleman, Phyllis R. Robinson, Tiffany M. Schmidt

**Affiliations:** 1 Department of Neurobiology, Northwestern University, Evanston, IL 60208, USA; 2 Interdisciplinary Biological Sciences Program, Northwestern University, Evanston, IL 60208, USA; 3 University of Maryland Baltimore County, Department of Biological Sciences, Baltimore, MD 21250, USA; 4 Section on Light and Circadian Rhythms (SLCR), National Institute of Mental Health, NIH, Bethesda, MD 20892, USA; 5 Department of Ophthalmology, Feinberg School of Medicine, Chicago, IL 60611, USA

**Keywords:** Retina, Vision, Phototransduction, Cascades, Signaling, GPCR, Melanopsin

## Abstract

Melanopsin is a visual pigment that is expressed in a small subset of intrinsically photosensitive retinal ganglion cells (ipRGCs). It is involved in regulating non-image forming visual behaviors, such as circadian photoentrainment and the pupillary light reflex, while also playing a role in many aspects of image-forming vision, such as contrast sensitivity. Melanopsin was initially discovered in the melanophores of the skin of the frog *Xenopus*, and subsequently found in a subset of ganglion cells in rat, mouse and primate retinas. ipRGCs were initially thought to be a single retinal ganglion cell population, and melanopsin was thought to activate a single, invertebrate-like G_q_/transient receptor potential canonical (TRPC)-based phototransduction cascade within these cells. However, in the 20 years since the discovery of melanopsin, our knowledge of this visual pigment and ipRGCs has expanded dramatically. Six ipRGC subtypes have now been identified in the mouse, each with unique morphological, physiological and functional properties. Multiple subtypes have also been identified in other species, suggesting that this cell type diversity is a general feature of the ipRGC system. This diversity has led to a renewed interest in melanopsin phototransduction that may not follow the canonical G_q_/TRPC cascade in the mouse or in the plethora of other organisms that express the melanopsin photopigment. In this Review, we discuss recent findings and discoveries that have challenged the prevailing view of melanopsin phototransduction as a single pathway that influences solely non-image forming functions.

## Abstract (precis)

**Summary:** This Review summarizes recent findings on the diversity of melanopsin phototransduction mechanisms described in subtypes of intrinsically photosensitive retinal ganglion cells and the conservation of these cascades across the evolutionary tree.

## Introduction

Across the animal kingdom, light drives myriad physiological changes and visual behaviors. Historically, light detection by the retina was thought to occur solely through the absorption of photons by rod and cone photoreceptors, producing an electrochemical signal that is relayed via a network of interneurons to the retinal ganglion cells (RGCs; see Glossary). RGCs are the output cells of the retina; they send axons to more than 40 brain regions to drive a diverse array of visual behaviors (Morin and Studholme, 2014). In the late 20th century, several key observations challenged this canonical model. For example, blind patients with degenerated rods and cones lacked visual perception but retained normal circadian photoentrainment (see Glossary) and light-evoked suppression of melatonin secretion (Czeisler et al., 1995; Lockley et al., 1997). Patients who had had their eyes removed lacked light-dependent effects on melatonin synthesis (Lockley et al., 1997), indicating that this light detection occurred in the eye. Likewise, mice genetically engineered to lack rods and cones showed functional circadian photoentrainment, pineal responses to light and pupillary light reflex (PLR; see Glossary) (Freedman et al., 1999; Lucas et al., 1999, 2001; Yoshimura and Ebihara, 1996). These studies suggested the existence of a third class of retinal photoreceptor. A subsequent study found that a small portion of RGCs in the mammalian retina express the photopigment melanopsin (gene symbol, *Opn4*), originally identified in *Xenopus laevis* and highly conserved across the animal kingdom (Provencio et al., 1998, 2000, 2002). Thus, melanopsin-expressing RGCs became a candidate for this third class of photoreceptor.

In 2002, researchers conclusively identified intrinsically photosensitive retinal ganglion cells (ipRGCs; see Glossary) as this novel third class of photoreceptor (Berson et al., 2002; Hattar et al., 2002). Like conventional RGCs, ipRGCs receive rod and cone input and project to several brain nuclei (Berson et al., 2002; Hattar et al., 2002, 2006; Schmidt et al., 2008; Schmidt and Kofuji, 2009; Zhao et al., 2014). However, unlike conventional RGCs, ipRGCs express melanopsin, and thus respond to light in the absence of rod and cone input (Hattar et al., 2002; Berson et al., 2002). ipRGCs depolarize in response to light with a slow and sustained response that persists from seconds to minutes (Hattar et al., 2002; Berson et al., 2002). In contrast, rods and cones hyperpolarize in response to light with millisecond precision (Hestrin and Korenbrot, 1990).

Intriguingly, melanopsin protein structure and its downstream signaling cascade in vertebrates more closely resembles the opsin structure/cascades found in invertebrate rhabdomeric photoreceptors (R-opsins; see Glossary) than those of the ciliary photoreceptors (C-opsins; see Glossary) found in vertebrates (Provencio et al., 1998, 2000; Koyanagi et al., 2005; Koyanagi and Terakita, 2008). The phototransduction cascade of rhabdomeric photoreceptors utilizes the G_q_ signaling pathway instead of the G_t_ pathway used by ciliary photoreceptors like rods and cones (Hardie, 2001, 2011; Palczewski, 2006). Photoreceptors belonging to the rhabdomeric lineage have a well-conserved phototransduction cascade: the G_q_ protein activates phospholipase C (PLC) to generate secondary messengers and target transient receptor potential (TRP) channels (see Glossary), resulting in depolarizing currents in the cell (Hardie, 2001, 2011). Indeed, early work on ipRGC phototransduction suggested that melanopsin signals exclusively through the G_q_ pathway (Graham et al., 2008; Hartwick et al., 2007; Perez-Leighton et al., 2011; Warren et al., 2006; Xue et al., 2011), although we now know that there may be some exceptions within the ipRGC population.

Initially, ipRGCs were thought to be a uniform population, targeting brain regions associated with non-image forming vision (see Glossary) and influencing subconscious visual behaviors (Berson et al., 2002; Hattar et al., 2002, 2006; Güler et al., 2008; Hatori et al., 2008). However, research has since uncovered multiple ipRGC subtypes: M1–M6. M1 ipRGCs are perhaps the best-characterized of the subtypes and were the first identified. They project to non-image forming brain regions to drive functions such as circadian photoentrainment and the PLR (reviewed in Aranda and Schmidt, 2021). M2–M6 ipRGCs primarily project to brain regions that play a role in conscious visual perception, though some subtypes do send minor projections to non-image forming brain regions (Hattar et al., 2006; Ecker et al., 2010; Estevez et al., 2012; Schmidt et al., 2014; Zhao et al., 2014; Stabio et al., 2018; Quattrochi et al., 2019; Sonoda et al., 2018; Huang et al., 2019). Non-image forming behaviors operate across sustained timescales, often integrating light information over hours and days, whereas visual perception requires high spatial and temporal resolution. Surprisingly, disruption of the slow and sustained melanopsin phototransduction cascade results in deficits in both M1-dependent, non-image forming behaviors as well as M2–M6-dependent visual perception (Wong et al., 2005; Gamlin et al., 2007; Do et al., 2009; Kankipati et al., 2010; Spoida et al., 2016; Yasin et al., 2017). Despite this diversity in structure and function of ipRGC subtypes, the melanopsin phototransduction cascade was long assumed to be similar across subtypes (Do et al., 2009; Spoida et al., 2016; Yasin et al., 2017). However, recent work suggests that melanopsin phototransduction pathways are optimized within individual ipRGC subtypes to drive their associated behaviors. In this Review, we will summarize the current state of knowledge on melanopsin protein structure and phototransduction, and how melanopsin signals in ipRGCs across multiple species.

Glossary **Ciliary photoreceptors**

Photoreceptors defined by the presence of modified cilia in their structure. Ciliary photoreceptors express visual pigments in stacked layers of membrane and are generally expressed in vertebrates.

**Circadian photoentrainment**

The entrainment of an organism's endogenous, internal circadian rhythms to the external day–night cycle. Organisms with endogenous activity periods greater or less than 24 h can be tuned to precisely 24 h via this entrainment.

**Contrast sensitivity**

The ability to distinguish an object from the background behind it. Cellular contrast sensitivity is the sensitivity of single cells to contrast. Behavioral contrast sensitivity is a measure of a behaving animal's ability to detect differences in contrast.

**Dermal phototaxis**

The bodily movement of an organism either towards (positive phototaxis) or away (negative phototaxis) from light in response to photoreception by the skin (derma).

**Extrinsic input**

Light input to a cell generated from another cell in the circuit. For example, ipRGCs receive ‘extrinsic’ light input relayed from rod and cone photoreceptors via retinal circuits.

**Hyperpolarization-activated, cyclic nucleotide-gated (HCN) channels**

Non-selective voltage-gated cation channels are encoded by four genes (*Hcn1*–*Hcn4*) and are principally operated by voltage. These channels open at hyperpolarized potentials and close upon depolarization. HCN channels can also be activated by cyclic nucleotides, such as cAMP, by shifting its activation curve to more depolarized voltages.

**Image-forming vision**

Visual functions that involve visual perception, identification and tracking of objects in the visual world. Examples of these functions include color vision, pattern recognition and contrast detection.

**Intrinsic input**

Light input to a cell generated cell autonomously. Melanopsin signaling within ipRGCs is considered intrinsic input.

**Inner plexiform layer (IPL)**

The retina is an organized structure divided into several layers; the IPL contains the synaptic connections between intermediate neurons, such as bipolar and amacrine cells, and the dendrites of RGCs. The IPL consists of ON and OFF sublaminas; this terminology was determined by the ON and OFF bipolar cell types terminating and making synaptic connections with ON and OFF RGCs, respectively.

**Intrinsically photosensitive retinal ganglion cells (ipRGCs)**

A class of photoreceptors that express the photopigment melanopsin, rendering them intrinsically photosensitive. In mice, there are six reported ipRGCs subtypes, M1–M6; however, in other organisms such as humans, M1–M4 have been identified.

**Light onset**

The start of a light stimulus (i.e. when light is turned on)

**Mesopic light**

Luminance ranging from ∼10–11 log photons cm^−2^ s^−1^. This an intermediate luminance between scotopic and photopic and is detected by rod and cone photoreceptors. For example, moonlight under the full moon is mesopic light.

**Non-image forming vision**

Light-driven behaviors or physiological functions that occur outside of conscious perception, including circadian photoentrainment, sleep, the pupillary light reflex and the effects of light on mood. ipRGCs project to brain regions involved in non-image forming vision; M1 ipRGCs are the major subtype driving these behaviors.

**Photopic light**

Luminance of ∼11 or greater log photons cm^−2^ s^−1^. Photopic light is detected by cone photoreceptors, which convey color information for visual perception. Daylight and standard indoor lighting are photopic.

**Pupillary light reflex (PLR)**

The reflexive constriction and dilation of the pupil to changes in environmental light. The pupil constricts more with brighter light.

**Retinal ganglion cells (RGCs)**

The output neurons of the retina that receive input relayed from the rods and cones via retinal circuits. RGCs project to several brain regions to influence both image-forming and non-image forming visual behaviors. Over 40 types of retinal ganglion cells (of which ipRGCs are a subset) have been identified in the mouse retina.

**Rhabdomeric photoreceptors**

Photoreceptors composed of stacks of microvilli that form a rod-like structure. Visual pigments are localized in these rod-like structures or rhabdoms. Rhabdomeric photoreceptors are generally found in invertebrates.

**Scotopic light**

Luminance of ∼10 or less log photons cm^−2^ s^−1^. Scotopic light is detected predominantly by rod photoreceptors. For example, starlight on a moonless night is scotopic light.

**Transient receptor potential (TRP) channels**

A family of non-selective cation channels found in the plasma membranes of cells. Opsins in rhabdomeric photoreceptors activate well-conserved G_q_ signaling pathways to open TRP channels. Melanopsin phototransduction cascades in some ipRGC subtypes open TRPC channels to depolarize the cell.

## The photopigment melanopsin: protein structure

Melanopsin is a canonical G-protein coupled receptor (GPCR), with seven transmembrane helices, an extracellular amino-terminus and an intracellular carboxy-tail (Terakita, 2005). Functional, light-sensitive melanopsin protein is covalently bound to a vitamin A-derived chromophore, 11*-cis* retinal (Walker et al., 2008). Retinal serves as an antagonist to opsin signaling, maintaining the inactive confirmation of the molecule until the chromophore absorbs a photon of light. Predictions from other visual pigments such as rhodopsin suggest that isomerization of the chromophore by light to all-*trans* retinal removes this antagonism and permits a conformational change in the opsin that allows for G-protein binding, activation and initiation of a downstream signaling cascade (Farrens et al., 1996).

Melanopsin shares greater sequence homology to R-opsins than to C-opsins. The insertion of eight amino acids in the third extracellular loop and an extended carboxy-tail in melanopsin – features not found in C-opsins – may influence G-protein binding and kinase activity, respectively, thus modulating melanopsin signaling (Provencio et al., 1998). The interaction of melanopsin with its chromophore is also reminiscent of R-opsins, as the visual pigment is suggested to be bi-stable – that is, capable of regenerating 11-*cis* retinal independently of external isomerases after photo-bleaching – or even tri-stable, with one signaling state and two silent states from which melanopsin can be activated by light (Koyanagi et al., 2005; Panda et al., 2005; Mure et al., 2007, 2009; Matsuyama et al., 2012; Emanuel and Do, 2015). In contrast, C-opsins release their chromophore upon isomerization and must be regenerated with new chromophore before re-initiating signaling (for a review, see Palczewski and Kiser, 2020). Such bi-stability may influence the sustained signaling properties of melanopsin (for a detailed discussion of R- versus C-opsins, see Porter et al., 2012). Unlike most C- and R-opsins, melanopsin is not confined to specialized membrane compartments, but is instead localized throughout the plasma membrane of the cell bodies and dendrites of ipRGCs at a lower concentration than is found in rods and cones, both of which likely contribute to the relatively lower sensitivity of melanopsin phototransduction (Hattar et al., 2002; Provencio et al., 2002; Belenky et al., 2003; Do et al., 2009; Do, 2019 but see Lee et al., 2019 and Sonoda et al., 2018).

**Fig. 1.**
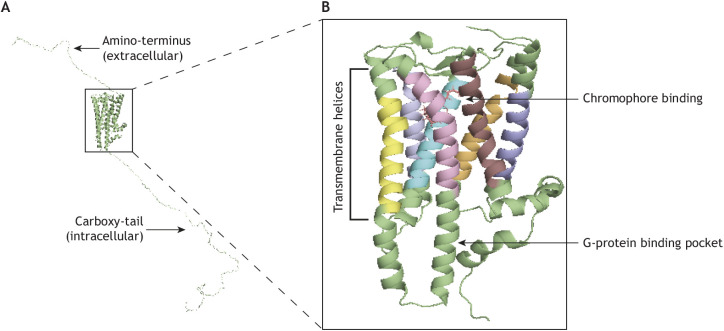
**Structure of mammalian Opn4 based on squid rhodopsin.** (A) 3D homology modeling of inactive mouse melanopsin based on a squid (*Todarodes pacificus*) rhodopsin template. Modeling was done in PyMOL (The PyMOL Moelcular Graphics System, v.1.2r3pre, Schrödinger, LLC). Mouse melanopsin boasts an exceptionally long carboxy-tail that heavily influences both activation and deactivation of melanopsin signaling. (B) An enhanced view of the transmembrane regions of mouse melanopsin, including chromophore binding. Each distinct transmembrane helix is represented in a different color. Upon isomerization of the chromophore by light, transmembrane movement of the transmembrane helices permits the binding of G-protein to intracellular loops two and three.

The crystal structure of melanopsin has yet to be elucidated. However, modeling of the three-dimensional melanopsin structure based on squid rhodopsin (an R-opsin), gives key insights to the structure–function relationship of the molecule, especially the role of the carboxy-tail (Fig. 1). Although phosphorylation of the carboxy-tail of many GPCRs is important for deactivation of signaling (see below), the carboxy-tail of mouse melanopsin (especially phosphorylation of sites on the proximal portion of the tail) is also required for appropriate activation of the molecule *in vitro* (Valdez-Lopez et al., 2020b). Furthermore, across species, the activation kinetics of melanopsin can be predicted by the number and location of phosphorylation sites on the proximal site of the carboxy-tail, suggesting conservation of carboxy-tail-dependent activation (Blasic et al., 2014; Valdez-Lopez et al., 2020b).

In contrast to the exceptionally fast signaling kinetics of rod and cone opsins, melanopsin phototransduction drives a very sustained light response in ipRGCs, with slower onset and offset kinetics than that of rods and cones (Hestrin and Korenbrot, 1990; Berson et al., 2002). In the context of the canonical role of melanopsin as a ‘non-image forming’ visual pigment, this seems advantageous; melanopsin signals sustained light information for the purposes of circadian photoentrainment, the PLR and other non-image forming light-dependent processes, such as mood (Hattar et al., 2003; Mrosovsky and Hattar, 2003; Lucas et al., 2003; Panda et al., 2003; Gooley et al., 2012; LeGates et al., 2012; Fernandez et al., 2018). These functions occur over the course of minutes or even hours, which parallels the slow kinetics of melanopsin phototransduction. However, melanopsin has also recently been implicated in visual perception, including in processes such as contrast sensitivity (see Glossary) and spatial discrimination (Dacey et al., 2005; Brown et al., 2010; Ecker et al., 2010; Schmidt et al., 2014; Allen et al., 2017; Sonoda et al., 2018; Allen, et al., 2019). These functions require much faster kinetics and higher resolution than previously measured for melanopsin signaling in the relevant ipRGC subtypes (M2–M6). The slow kinetics of melanopsin are able to influence these aspects of image-forming vision (see Glossary) by altering the intrinsic properties of the ipRGCs according to sustained background light levels (Sonoda et al., 2018). Therefore, this single photopigment influences a broad range of light-evoked behaviors and physiological effects. Emerging work indicates that melanopsin phototransduction acts through different signaling cascades across ipRGC subtypes, which may underlie this diversity of melanopsin-dependent functions in mammals and other vertebrates (Sonoda et al., 2018; Jiang et al., 2018; Graham et al., 2008; Xue et al., 2011; Perez-Leighton et al., 2011).

## ipRGC subtypes

**Fig. 2.**
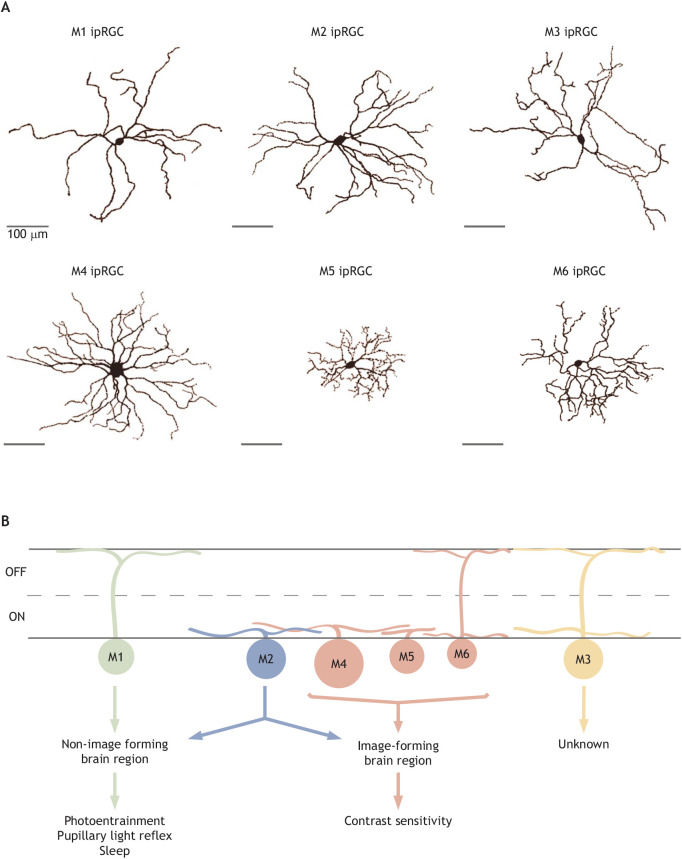
**Overview of ipRGC subtype diversity.** (A) Tracings of single M1–M6 ipRGCs. The properties of these cell types are summarized in Table 1. (B) A simplified schematic of M1–M6 stratification in the ON and OFF sublamina of the inner plexiform layer (IPL) depicting downstream targets. The projections of M3 have not been well-studied; however, they may innervate the superior colliculus (Zhao et al., 2014). The M1–M5 dye-filled cells were collected in the Schmidt laboratory, and the M6 morphology is reproduced from Quattrochi et al. (2019), with permission.

ipRGCs differ in their morphology, brain projections, behavioral roles, rod/cone input and melanopsin phototransduction pathways (Fig. 2; Table 1). Below and in Table 1, we briefly summarize the properties and functions of each ipRGC subtype (for detailed reviews, see Sonoda and Schmidt, 2016; Lazzerini Ospri et al., 2017; Do, 2019; Sondereker et al., 2020; Aranda and Schmidt, 2021). Note that the vast majority of this work has been performed in mouse, and studies in other species will be necessary to determine to what extent these features of the phototransduction cascade generalize across species.

**Table 1.**
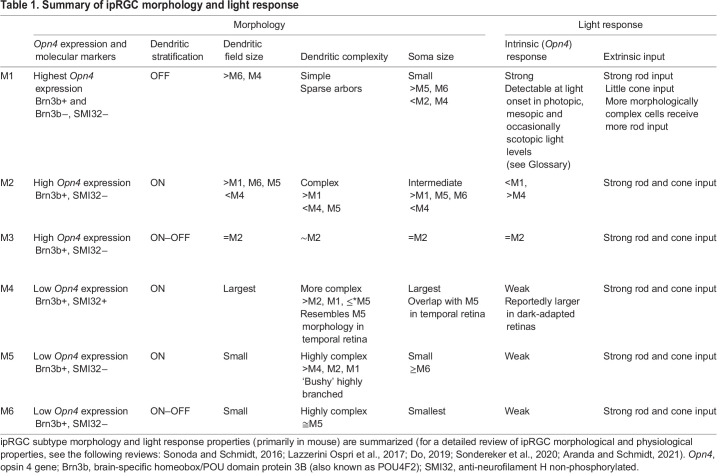
Summary of ipRGC morphology and light response

M1 ipRGCs project to non-image forming brain regions such as the suprachiasmatic nucleus (SCN) and olivary pretectal nucleus (OPN), among others, and are critical for non-image forming visual functions such as circadian photoentrainment, the PLR, sleep induction by light and the effects of exposure to irregular light/dark cycles (‘aberrant light’) on learning and mood (Hattar et al., 2002, 2006; Gooley et al., 2003; Baver et al., 2008; Panda et al., 2002; Ruby et al., 2002; Hatori et al., 2008; Göz et al., 2008; Güler et al., 2008; Chew et al., 2017; Lucas et al., 2003; Altimus et al., 2008; Lupi et al., 2008; Rupp et al., 2019; Chen et al., 2011; LeGates et al., 2012; Fernandez et al., 2018). These cells stratify in the OFF sublamina of the inner plexiform layer (IPL; see Glossary), express the highest levels of melanopsin, and have the smallest and simplest dendritic arbors (Fig. 2; Baver et al., 2008; Berson et al., 2002, 2010; Hattar et al., 2002, 2006; Viney et al., 2007; Schmidt and Kofuji, 2009; Müller et al., 2010; Ecker et al., 2010; Estevez et al., 2012). M3 ipRGCs have larger, more complex dendritic arbors, are bistratified and express the next highest levels of melanopsin (Fig. 2; Schmidt and Kofuji, 2011). M2 ipRGCs are ON stratified and have dendritic arbor size/complexity similar to that of M3 ipRGCs but lower melanopsin expression than M3 cells. M4 ipRGCs have the largest somata of all ipRGCs and the largest dendritic arbors, which ramify in the ON sublamina (Fig. 2; Ecker et al., 2010; Estevez et al., 2012; Schmidt et al., 2014; Bleckert et al., 2014; Sonoda et al., 2020). M5 and M6 ipRGCs have small, bushy, highly branched dendritic arbors and are ON (M5) or bistratified (M6) (Fig. 2; Ecker et al., 2010; Stabio et al., 2018; Sonoda et al., 2020; Quattrochi et al., 2019). M4–M6 ipRGCs express levels of melanopsin so low that they are undetectable with immunohistochemistry for the melanopsin protein. Non-M1 (M2–M6) ipRGCs collectively project to image-forming brain regions where they influence pattern vision functions such as contrast sensitivity (Ecker et al., 2010; Estevez et al., 2012; Zhao et al., 2014; Schmidt et al., 2014; Stabio et al., 2018; Quattrochi et al., 2019). It is worth noting that M2 ipRGCs also send projections to the SCN, a non-image forming brain region (Baver et al., 2008), and their function in circadian photoentrainment is unknown. M4 ipRGCs also send projections to the ventral lateral geniculate nucleus (vLGN) and have been implicated in the modulation of mood circuits (Huang et al., 2019).

## Phototransduction in the retina

**Fig. 3.**
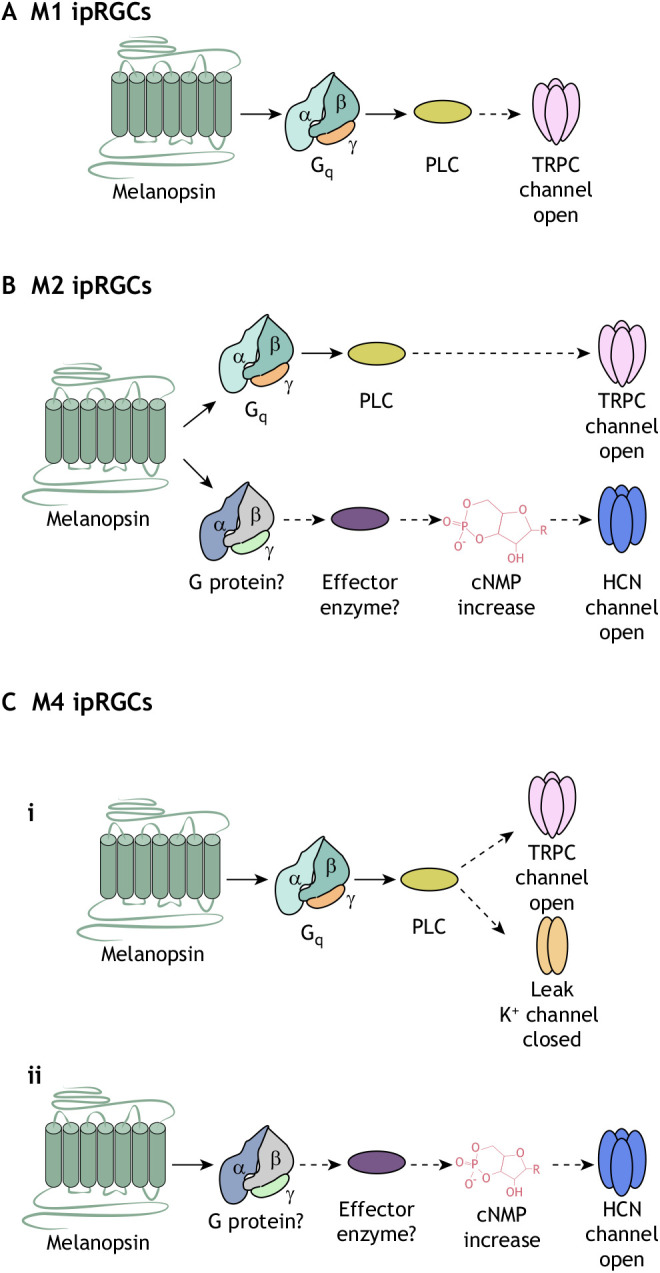
**Melanopsin phototransduction cascade models in ipRGCs.** Light activates melanopsin, initiating a biochemical cascade resulting in the depolarization of ipRGCs. Diverse melanopsin phototransduction cascades exist across ipRGC subtypes. Depicted are the current phototransduction models for three ipRGC subtypes. (A) In M1 ipRGCs, melanopsin signals through G_q_ to target TRPC channels (Warren et al., 2006; Graham et al., 2008; Xue et al., 2011; Jiang et al., 2018; Sonoda et al., 2018). (B) Similarly to M1 cells, M2 ipRGCs activate G_q_ to modulate TRPC channels, yet unlike M1 ipRGCs, M2 cells in parallel signal through an unknown G-protein to open HCN channels (Jiang et al., 2018; Perez-Leighton et al., 2011). (C) There are two proposed models for M4 ipRGC phototransduction (Sonoda et al., 2018; Jiang et al., 2018). (i) Sonoda et al. (2018) propose that potassium leak channels are the major phototransduction target in M4 ipRGCs with a minor role played by TRPC channels. (ii) HCN channels are the major phototransduction target in M4 cells (Jiang et al., 2018). HCN channels are primarily opened by hyperpolarization and closed by depolarization; however, in this model, HCN channels are activated by cyclic nucleotides. The structure of a cyclic nucleotide is shown above, in which the R represents the nitrogenous base (adenine or guanine) bonded to the sugar phosphate part. Further inquiry is required to explain the opposing melanopsin phototransduction models in M4 ipRGCs. cNMP, cyclic nucleotide monophosphate; HCN, hyperpolarization-activated cyclic nucleotide-gated; PLC, phospholipase C; TRPC, canonical transient receptor potential.

As discussed above, since their discovery in 2002, a total of six ipRGC subtypes have been identified, but much of the study of phototransduction pathways has been confined to M1 cells (primarily in mice). However, the heterogeneity in the melanopsin photoresponse kinetics and size among ipRGC subtypes, coupled with the distinct effects of ipRGC subtypes on various visual behaviors, raises the question of whether melanopsin might use diverse phototransduction cascades across ipRGC subtypes. Indeed, recent research strongly supports a diversity of melanopsin phototransduction in mammalian ipRGC subtypes from scotopic to photopic luminance (see Glossary; Emanuel et al., 2017; Sonoda et al., 2018; Lee et al., 2019). Below, we review the emerging evidence for diversity of the melanopsin phototransduction cascade across ipRGC subtypes studied in the retina (Fig. 3).

### M1 phototransduction cascade

**Fig. 4.**
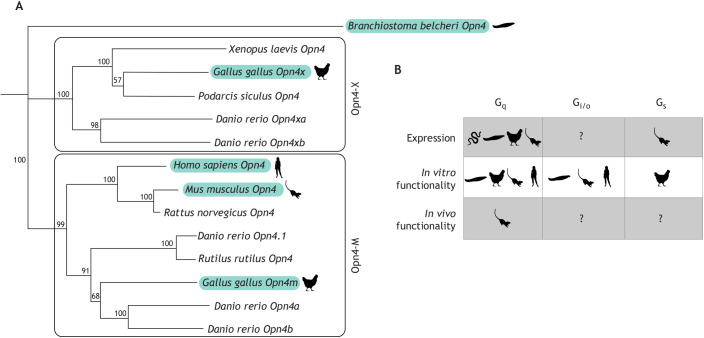
**Phylogeny and current understanding of melanopsin phototransduction across species.** (A) Phylogenetic analysis of the melanopsin protein sequence across multiple organisms using the neighbor-joining method (Saitou and Nei, 1987). Amino acid sequences of 14 melanopsin genes expressed in 9 different organisms (UniProt, Table S1) were aligned using MUSCLE alignment (Edgar, 2004) and compiled into a tree using the neighbor-joining method within Geneious Prime (Geneious Prime v.2019.1.1.; https://www.geneious.com). Amphioxus (*Branchiostoma belcheri*) melanopsin is used as an outgroup. Branches are labeled with percent confidence levels based on 100 bootstrap replicates. Only the melanopsin(s) of highlighted species have been studied in the context of phototransduction. (B) A summary of the current knowledge of the cognate G-proteins of melanopsin across multiple organisms. ‘Expression’ denotes either expression of protein or expression of mRNA transcripts that co-localize with melanopsin. Expression and *in vitro* assessment of G-protein activation has been defined across multiple organisms, whereas *in vivo* work has been done exclusively in the mouse.

The M1 melanopsin phototransduction cascade is the best characterized of all ipRGC subtypes and most similar to what has been found *in vitro* (for information on melanopsin signaling *in vitro*, see Box 1 and Fig. 4B). In M1 cells, melanopsin signals through a G_q_ cascade to modulate canonical TRP (TRPC) channels and elicit a light response (Fig. 3; Warren et al., 2006; Graham et al., 2008; Xue et al., 2011; Jiang et al., 2018; Sonoda et al., 2018). The signaling cascade begins with the light activation of melanopsin. Although melanopsin is thought to signal through a heterotrimeric G protein from the G_q_ protein family, the exact G protein subunit or subunits are unknown (Warren et al., 2006; Graham et al., 2008; Xue et al., 2011; Chew et al., 2014; Jiang et al., 2018). Mice with deletions of G_q_, G_11_, G_14_ or G_15_, either individually or in pairs, have pupil constriction and circadian behaviors that are comparable to those of wild-type mice (Chew et al., 2014). However, acute knockdown of G_q_, G_11_ and G_14_ mRNA in combination, but not individually, results in severe deficits in pupil constriction (Hughes et al., 2015). This suggests that melanopsin may be capable of activating all of these G_q_ family members in M1 ipRGCs. Recordings from dissociated M1 ipRGCs or M1 ipRGCs in *ex vivo* retinas support melanopsin signaling through G_q_ (Graham et al., 2008; Xue et al., 2011; Jiang et al., 2018). Specifically, cellular recordings performed on M1 cells reveal a reduction in photocurrent when the cells are exposed to a specific G_q_/G_11_-class G protein antagonist or missing G_q_ subunits (Graham et al., 2008; Xue et al., 2011; Jiang et al., 2018). At first glance, the behavioral data and the cellular recordings appear to be in conflict, yet *in vitro* work (Bailes and Lucas, 2013; Kankanamge et al., 2018) demonstrates that melanopsin can recognize multiple G proteins. Therefore, it is possible that, under different conditions, melanopsin can interact with other G proteins in the absence of G_q_ subunits.

**Box 1. Melanopsin signaling *in vitro***

Initial work to elucidate the melanopsin signaling cascade involved heterologous expression of the opsin *in vitro.* Melanopsin excited by blue light is capable of activating transducin *in vitro*, demonstrating that it could functionally activate a G-protein (Newman et al., 2003). However, transducin is not expressed in ipRGCs, and thus could not be the cognate G-protein of melanopsin. Subsequently, members of the G_q_ family of G-proteins were established as likely candidates owing to the loss of melanopsin signaling after inhibition of G_q_ and its downstream signaling component PLCβ (Isoldi et al., 2005; Panda et al., 2005; Qiu et al., 2005). Additionally, the higher sequence homology of melanopsin to R-opsins that utilize G_q_ also gave credence to the involvement of this pathway in melanopsin signaling (Terakita et al., 2008). Of the G_q_ family G-proteins, G_q_, G_11_ and G_14_ are all capable of signaling via melanopsin activation (Hughes et al., 2015).

Downstream of PLCβ activation by G_q_, phosphatidylinositol (4,5)-bisphosphate (PIP2) is hydrolyzed into diacylglycerol (DAG) and cytosolic inositol 1,4,5 trisphosphate (IP3). Both of these second messengers can then serve various functions within the cell. The extent to which they are each activated by melanopsin in ipRGC subtypes has yet to be determined. However, recent work *in vitro* implicates both activation of PLCβ4 and an increase in DAG in human melanopsin signaling (Krzysztynska-Kuleta et al., 2021). In *Drosophila* rhodopsin phototransduction, TRP channels (non-selective cation channels) are opened by the G_q_ pathway. Thus, canonical TRP (TRPC) channels became prime candidates for melanopsin phototransduction in vertebrates. Blockade of TRPCs reduces melanopsin-mediated calcium release as well as the intrinsic light response *in vitro* and *in vivo* (Warren et al., 2006; Hartwick et al., 2007; Sekaran et al., 2007). TRPC subtypes 6 and 7 (TRPC6/7) are expressed in ipRGCs, suggesting that these channels may be involved in melanopsin phototransduction, most likely in heteromeric conformations (Warren et al., 2006; Hartwick et al., 2007; Sekaran et al., 2007). However, the source of the melanopsin-mediated calcium increase within ipRGCs remains debated. Both release of internal calcium stores via IP3 (Melyan et al., 2005; Kumbalasiri et al., 2007) and influx of extracellular calcium via TRPC channels (Hartwick et al., 2007; Sekaran et al., 2007) have been reported.

Ultimately, the majority of *in vitro* work involving melanopsin signal transduction has focused on G_q_ signaling initiating a downstream cascade leading to the release of calcium either from internal or external calcium stores. But recent research reaffirms that melanopsin may indeed be a promiscuous GPCR, capable of utilizing multiple G-protein cascades, including G_i_ and G_βγ_ signaling (Peirson et al., 2007; Bailes and Lucas, 2013; Kankanamge et al., 2018).

Once melanopsin activates G_q_, this protein then proceeds to activate PLC (Graham et al., 2008; Xue et al., 2011; Jiang et al., 2018). In M1 cells, PLCβ4 is the PLC isoform associated with melanopsin phototransduction (Graham et al., 2008; Xue et al., 2011; Jiang et al., 2018). M1 cells have deficits in their light response when PLCβ4 is either pharmacologically blocked or genetically ablated (Graham et al., 2008; Xue et al., 2011; Jiang et al., 2018). In M1 cells, the phototransduction cascade results in the modulation of TRPC channels (Warren et al., 2006; Graham et al., 2008; Xue et al., 2011; Perez-Leighton et al., 2011; Sonoda et al., 2018; Jiang et al., 2018). The melanopsin photocurrent reverses near 0 mV and is abolished by blocking or eliminating TRPC3/6/7 channels, suggesting that these are indeed the major target of melanopsin phototransduction in M1 ipRGCs (Warren et al., 2006; Graham et al., 2008; Xue et al., 2011; Perez-Leighton et al., 2011; Sonoda et al., 2018; Jiang et al., 2018).

### M2 phototransduction cascade

As in M1 cells, the melanopsin photocurrent in M2 ipRGCs is also reduced by knockout or blockade of TRPC channels, suggesting some conservation of the melanopsin phototransduction cascade between M1 and M2 ipRGCs (Fig. 3; Jiang et al., 2018; Perez-Leighton et al., 2011). Some M2 cells have a fast and slow component in their melanopsin photocurrent in response to bright light stimulation, whereas other cells lack a clear transient (fast) component (Jiang et al., 2018). This fast component is abolished by adult knockout of G_q_, G_11_ and G_14_ or by knockout of PLCβ4. It is also abolished by knockout of TRPC3 and TRPC6 or pharmacological inhibition of TRPC channels. Moreover, the overall amplitude of the response is reduced by more than 50% following knockout of PLCβ4 or TRPC3/6, or pharmacological inhibition of TRPC channels, indicating that the TRPC pathway plays a critical role in M2 melanopsin phototransduction (Jiang et al., 2018), as suggested by previous work knocking out single TRPC6 subunits (Perez-Leighton et al., 2011).

Intriguingly, hyperpolarization-activated, cyclic nucleotide-gated (HCN) channels (see Glossary) are implicated in driving the non-TRPC component of the M2 light response (Jiang et al., 2018), as activation of cyclic nucleotides, in the form of cyclic nucleotide monophosphate (cNMP), is sufficient to depolarize M2 ipRGCs. Moreover, the photocurrent of TRPC3/6-knockout M2 cells is abolished upon exposure to an HCN antagonist (Jiang et al., 2018). The melanopsin photocurrent in M2 cells is also inhibited after expression of a dominant-negative mutation of the HCN2 subunit, suggesting that melanopsin targets HCN channels (Jiang et al., 2018). Based on these findings, Jiang and colleagues proposed that melanopsin leads to the opening of both TRPC and HCN channels in M2 cells.

### M4 phototransduction cascade

Melanopsin signaling in M4 ipRGCs, also called ON-sustained alpha RGCs, enhances cellular and behavioral contrast sensitivity in mice (Schmidt et al., 2014; Sonoda et al., 2018). Currently, there are two very distinct models for M4 ipRGC melanopsin phototransduction (Fig. 3; Sonoda et al., 2018; Jiang et al., 2018). The first model, published by Sonoda et al. (2018), states that potassium leak channels are the major phototransduction target in M4 ipRGCs and that TRPC channels have a small role in the signaling cascade. The second model, published by Jiang et al. (2018), proposes that HCN channels are the major phototransduction target in M4 cells and that TRPC channels are not part of this cascade. Below, we highlight the evidence for both models.

#### Model one: potassium leak channels are the major M4 phototransduction target

Sonoda et al. (2018) propose that M4 ipRGCs signal through G_q_ to close potassium leak channels and open TRPC channels to depolarize the cell (Fig. 3). In this work, M4 cells were identified as having large somata and an ON-sustained response to light and confirmed for immunoreactivity to SMI-32 (see Table 1; Schmidt et al., 2014). The authors showed that the melanopsin photocurrent was abolished by application of a G_q_ inhibitor or by a PLC inhibitor in different preparations. Activation of the G_q_ cascade is sufficient to drive a melanopsin-like response in M4 cells and rescue cellular deficits in contrast sensitivity in melanopsin-null M4 cells. Collectively, these data suggest that M4 ipRGCs use G_q_-mediated melanopsin phototransduction. Sonoda et al. (2018) next measured the reversal potential and current–voltage (I–V) relationship of the melanopsin photocurrent in M4 ipRGCs. They found that the M4 photocurrent had a negative slope and reversed near −90 mV, the equilibrium potential for potassium. These findings suggest that melanopsin phototransduction drives closure (due to the negative slope of the I–V relationship) of potassium channels (due to the reversal potential of −90 mV). Light stimulation with either scotopic or photopic light resulted in an increased input resistance and increased excitability in M4 cells. This further supports the model that melanopsin phototransduction closes potassium leak channels, because increased input resistance and cellular excitability would occur following potassium leak channel closure, but not after opening of a non-specific cation channel such as TRPC or HCN. The increase in input resistance is amplified in bright light in TRPC3/6/7-knockout M4 cells, suggesting a small but significant contribution of TRPC channels to melanopsin phototransduction in bright light. Collectively, these data indicate that melanopsin phototransduction is activated even under very dim conditions in M4 ipRGCs, and that melanopsin phototransduction acts through G_q_ pathways to close potassium channels.

#### Model two: HCN channels are the major phototransduction target in M4 cells

An alternative model for M4 phototransduction was proposed by Jiang et al. 2018 (Fig. 3). In this model, melanopsin phototransduction acts to open HCN channels but does not act on either TRPC or potassium channels. These authors reported no change in M4 melanopsin photocurrent in genetic models lacking TRPC3/6, G_q_, G_11_, G_14_ or PLCβ4, or in M4 cells with pharmacological inhibition of TRPC channels. Instead, they propose that the major phototransduction target in M4 cells is HCN channels. Their work suggests that increased cyclic nucleotide concentration is sufficient to evoke a current in M4 ipRGCs. Moreover, Jiang et al. (2018) report that the photocurrent from M4 cells lacking TRPC channels is eliminated by an HCN antagonist. To further support this, animals lacking TRPC channels were injected with a virus carrying a dominant-negative mutation of the HCN2 subunit, as discussed for M2 cells above. As in M2 ipRGCs, the light response of M4 cells from these animals is abolished. From these experiments, Jiang and colleagues proposed that the major phototransduction target in M4 ipRGCs is HCN channels.

#### Discrepancies between these models

As discussed above, there are several differences between the models proposed by Sonoda et al. (2018) and Jiang et al. (2018), and the two models are difficult to reconcile. Indeed, if melanopsin is opening HCN channels (which are non-selective cation channels) in M4 ipRGCs, the I–V curve of the photocurrent should have a positive slope (indicating channel opening) and reverse between −25 and −40 mV (reviewed in Biel et al., 2009). Yet, Sonoda and colleagues report a negative slope I–V relationship (indicating closure of the phototransduction channel) that reverses at the potassium equilibrium potential. Moreover, opening of an HCN channel would result in a decrease in input resistance in the presence of light, and yet Sonoda and colleagues report a light-evoked increase in input resistance, which is again consistent with potassium channel closure in response to light stimulation. Jiang and colleagues clearly show loss of the melanopsin photocurrent in the presence of the HCN antagonist ZD7288. However, ZD7288 has been shown to have nonspecific effects on other ion channels (Felix et al., 2003; Do and Bean, 2003; Sánchez-Alonso et al., 2008; Wu et al., 2012). Jiang et al., 2018 also reported a loss of melanopsin phototransduction in M4 ipRGCs following dominant-negative HCN2 expression.

The studies also reached opposing conclusions about the contribution of the G_q_ pathway. Sonoda et al. used pharmacological and chemogenetic means to show that G_q_ is both necessary and sufficient for melanopsin-dependent effects in M4 ipRGCs, whereas Jiang et al. demonstrated no effect of genetic removal of G_q_ family subunits. The two studies used slightly different recording conditions, mouse lines and approaches to reach their respective conclusions. For example, Jiang and colleagues used soma size and anatomical features of M4 ipRGCs to identify this cell type, whereas Sonoda et al. used physiological, morphological and immunohistochemical techniques to confirm the identity of M4 ipRGCs. As M4 ipRGCs have widely varying anatomical features across the retina (Sonoda et al., 2020), it is possible that the two studies were recording from different ipRGC populations. Opening of an HCN channel versus closure of a potassium channel would result in opposing, and therefore substantially different, changes in M4 cellular and photocurrent properties. Undoubtedly, future research is urgently needed to resolve this discrepancy and explain its origins.

## Deactivation

Unlike initiation of the melanopsin phototransduction cascade, research into the deactivation of the light response has been performed almost exclusively *in vitro* and therefore not in specific ipRGC subtypes*.* The melanopsin carboxy-tail serves as a site for phosphorylation, allowing subsequent binding of β-arrestin 1 and -2. *In vitro*, both arrestins are capable of terminating signaling, whereas *in vivo* it is suggested that β-arrestin 2 terminates signaling whereas β-arrestin 1 induces recycling of the visual pigment (Blasic et al., 2012; Cameron and Robinson, 2014; Mure et al., 2018). Compared with the rod and cone opsins, mouse melanopsin has an exceptionally long carboxy-tail (Fig. 1), with a total of 38 putative phosphorylation sites within the tail. This distinction from rod and cone opsins has prompted much research on the role this region plays in the deactivation of melanopsin. Six phosphorylation sites on the mouse melanopsin carboxy-tail (S388, T389, S391, S392, S394 and S395, together known as P-II) have been identified as necessary for proper deactivation of the molecule *in vitro* (Blasic et al., 2014). Interestingly, these six sites alone are not sufficient for normal melanopsin deactivation. Instead, additional amino acid phosphorylation events either upstream or downstream of the P-II cluster are also required, although there appears to be flexibility in the precise residues phosphorylated to initiate deactivation (Valdez-Lopez et al., 2020a). Questions still remain about the function of the distal region of the carboxy-tail, as it is not required for appropriate deactivation of melanopsin signaling *in vitro* (Valdez-Lopez et al., 2020a). However, loss of the distal tail produces faster deactivation than observed for wild-type melanopsin, suggesting that this region may have a role in extending deactivation, possibly by sterically hindering the access of signaling cascade components (Mure et al., 2016).

The role of the carboxy-tail in the deactivation of melanopsin *in vivo* is an emerging field of study. Mutation of all 38 putative phosphorylation sites on the melanopsin carboxy-tail to non-phosphorylatable residues results in major prolongation of the PLR: constriction of the pupil lasts up to 45 min post light stimulus in these mutant animals, indicating prolonged firing of ipRGCs and a critical role for phosphorylation-dependent deactivation of melanopsin in this behavior, although other non-image forming behaviors are not significantly impacted by these mutations (Somasundaram et al., 2017). Similarly, mutation of nine residues within the regions shown to be necessary and sufficient for appropriate deactivation of melanopsin *in vitro* produces a similar extension of the PLR, reflecting a prolonged deactivation of melanopsin *in vivo* (Mure et al., 2016). Additionally, in rat, light-dependent phosphorylation and de-phosphorylation of serines 381 and 398 significantly impacts the influx of calcium into the cell after light stimulation (Fahrenkrug et al., 2014). The deactivation of melanopsin clearly has important implications for the functioning of the photopigment and its impact on non-image forming behaviors. More work in this area – in conjunction with a greater understanding of differences in ipRGC subtype-specific activation of melanopsin – will be crucial to reveal a full picture of melanopsin signaling *in vivo.*

## Melanopsin signaling cascades in other organisms

Melanopsin expression is conserved across many species, from the chordate amphioxus to humans. Research in this area has focused on the evolution of melanopsin, its localization and expression both in the retina and extra-ocular regions, the impact of melanopsin signaling on a variety of behaviors, and the role of melanopsin-expressing cells in various behavioral and physiological circuits (for relevant reviews, see Rollag et al., 2003; Hankins et al., 2008; Bailes and Lucas, 2010; Peirson et al., 2009; Davies et al., 2010; Hatori and Panda, 2010; Schmidt et al., 2011).

It is important to note that vertebrate melanopsin is expressed from two distinct melanopsin genes: *Opn4m* (expressed in both mammalian and non-mammalian vertebrates) and *Opn4x* (expressed only in non-mammalian vertebrates) (Bellingham et al., 2006; Fig. 4). An evolutionary analysis of melanopsin from many organisms suggests that the protein may be capable of binding and activating both G_q_ and G_i/o_, and that melanopsin evolution (Fig. 4), particularly in the sequence of the second and third intracellular loops that make up the G-protein binding pocket, may have been driven by the specific signaling cascade components expressed within melanopsin-expressing cells (Fig. S1; Borges et al., 2012). Alignment of the G-protein binding domain of melanopsins from many species shows high sequence divergence at this region, and also suggests that different melanopsins may be capable of activating different G-proteins (Fig. S1; Davies, Hankins, and Foster, 2010). Even mouse melanopsin is known to have both short and long splice isoforms (Pires et al., 2009). Although differential expression of these isoforms may regulate distinct melanopsin-mediated behaviors (Jagannath et al., 2015), their precise roles remain to be determined. A comprehensive understanding of melanopsin in a wide variety of organisms is necessary to truly understand the evolutionary and functional consequences of G-protein specificity and the phototransduction cascade activated by this conserved visual pigment. Below, we discuss research focusing on the phototransduction cascade activated by melanopsin expressed across various species.

### Non-vertebrate chordates

The non-vertebrate chordate amphioxus (*Branchiostoma brancheri*) expresses melanopsin in its neural tube (Koyanagi et al., 2005). This melanopsin shows similar spectral tuning to vertebrate melanopsin, its expression co-localizes with G_q_, and it is capable of signaling via G_q_ and G_i/o_ *in vitro* (Fig. 4)*.* The involvement of PLC and IP3 in the signal transduction cascade of amphioxus melanopsin has been established *in vivo* (Koyanagi et al., 2005; Terakita et al., 2008; Gomez et al., 2009; Nasi and del Pilar Gomez, 2009; Angueyra et al., 2012; Bailes and Lucas, 2013). The expression of melanopsin in amphioxus suggests a possible ancestral lineage that ultimately led to the evolution of melanopsin in vertebrates. In addition, melanopsin expression in amphioxus (and possibly other invertebrates) may provide key insights to melanopsin phototransduction in vertebrates.

### Frogs

In frog (*X. laevis*) melanophores (the cells in which melanopsin was initially discovered), a key function for light activation of this melanopsin is melanosome dispersion, a process that produces coloration changes for both camouflage and social interactions. Stimulation of melanopsin in these cells initiates a cascade in which PLC and IP3/DAG are activated, subsequently activating protein kinase C, which is required for the dispersion of the melanosomes (Isoldi et al., 2005). In contrast, in the mouse retina, where stimulation of melanopsin by light produces a cellular depolarization, the downstream effects of PLCβ remain unclear. A difference in cascade beyond PLC activation is plausible, as melanopsin elicits quite different functional consequences between ipRGCs and frog melanophores; in fact, as described above, melanopsin may activate multiple cascades even within a single organism.

### Birds

Much work on melanopsin phototransduction outside of mice has been conducted in chicks (*Gallus gallus*). Opn4 mRNA is highly enriched in the chick retina and pineal gland, as well as being detected in various regions involved in visual processes, such as the optic tectum (Bailey and Cassone, 2005). Two distinct melanopsin genes and five total isoforms of the visual pigment are expressed in the chick pineal gland, which plays a major role in melatonin release and circadian rhythms (Torii et al., 2007). Analysis of the sequence of pineal melanopsin in chick suggests that it is capable of activating a G_q_ cascade, particularly through G_11_, which is expressed in the pineal gland and been shown to drive phase shifts of cultured pineal cells *in vitro* (Kasahara et al., 2002; Torii et al., 2007).

In the chick retina, both Opn4m and Opn4x transcripts can also be detected (Díaz et al., 2014). Opn4m transcripts are detected prior to Opn4x during development, and both are present in conjunction with G_q_ mRNA prior to the development of other G-protein transcripts such as transducin (Verra et al., 2011). Thus, melanopsin is hypothesized to activate a G_q_ cascade in the chick. In support of this hypothesis, inhibition of PLC and blockage of TRP channels (both downstream components of the G_q_ cascade) suppress the effect of light on melanopsin-mediated physiology *in vitro* (Fig. 4; Contín et al., 2006). Furthermore, downstream effectors PIP2, IP3 and DAG, as well as kinases DAGK, PIK and PIPK are all implicated in the ipRGC light response in chick, further supporting a melanopsin–G_q_ cascade (Fig. 4; Contín et al., 2010; Díaz et al., 2014).

These results suggest that melanopsin activates a G_q_ cascade in chick, especially during retinal development, as many of these studies employ early embryonic cell cultures. However, both chick Opn4x and Opn4m are also able to activate G_s_, as seen via an increase in cAMP reporter activity *in vitro* (Fig. 4; Bailes and Lucas, 2013). This suggests that melanopsin G-protein promiscuity may exist in the chick retina, as is thought to be the case in mice.

### Reptiles

Although reptile melanopsin remains less studied in comparison to that of other organisms, the signaling cascade(s) of reptile melanopsin have recently begun to be investigated. In the sea snake *Aipysurus laevis*, which demonstrates dermal phototaxis (see Glossary), Opn4x and genes coding for G_q_ family proteins (specifically G_11_) as well as PLCβ are expressed in the skin, suggesting that this melanopsin is functional and plays a role in driving dermal phototaxis (Fig. 4; Crowe-Riddell et al., 2019). Further study of reptiles may offer novel insights into melanopsin phototransduction due to the great variations in environment, lifestyle and behaviors of these organisms.

### Primates

Of course, one of the ultimate goals of the study of melanopsin is to understand melanopsin function in human ipRGCs, behavior and well-being. Primate and mouse ipRGCs show many similarities, including dendritic stratification and projections to many brain targets, such as the SCN, OPN and LGN (Hannibal et al., 2004; Dacey et al., 2005; Jusuf et al., 2007; Hannibal et al., 2014). Three types of human ipRGCs have been identified by their signaling properties, which correspond highly to three of the six known mouse ipRGC subtypes (Mure et al., 2019). In humans, ipRGCs and melanopsin have been implicated in the PLR as well as circadian photoentrainment and melatonin release, as they are in mice (Gamlin et al., 2007; Gooley et al., 2012; Ho Mien et al., 2014; Prayag et al., 2019). Additionally, mouse ipRGCs demonstrate a degree of molecular similarity to primate ipRGCs despite marked differences in other RGC types across both species, making the study of ipRGCs in the mouse a useful and important starting point for understanding human ipRGCs (Peng et al., 2019).

Like the mouse, human melanopsin is primarily expressed in the ganglion cell layer of the retina (Provencio et al., 2000). Study of human melanopsin suggests the possibility of melanopsin/G-protein promiscuity, as in mice. Melanopsin-dependent increases in intracellular calcium as well as decreases in cAMP have been observed in humans, supporting the coupling of both G_q_ and G_i_ G-proteins to human melanopsin (Fig. 4; Bailes and Lucas, 2013). Human melanopsin signaling through G_q_ is also supported by *in vitro* work demonstrating activation of PLCβ4 and an increase in DAG in an HEK293 cell line stably expressing human melanopsin (Krzysztynska-Kuleta et al., 2021). In contrast, however, other studies of human melanopsin have demonstrated that pharmacological block of G_i_ does not impair melanopsin signaling (Melyan et al., 2005). Although differences in experimental design may produce such a discrepancy, there is clearly much to be understood about the human melanopsin signaling cascade. Further study on melanopsin in humans and other organisms will provide greater insight into the role of this highly conserved pigment in behavior, physiology and health.

## Conclusions

At one time, the homology and similarities of melanopsin to invertebrate rhabdomeric visual pigments strongly implicated G_q_ and PLCβ4 as the G-protein and downstream effector involved in melanopsin signaling within ipRGCs. Early *in vitro* and *in vivo* work examining the melanopsin phototransduction cascade corroborated these ideas. However, the role of other effectors in the cascade – such as PIP2, IP3 and DAG – as well as how the pathway ultimately opens TRP channels has never been fully elucidated. Proposed mechanisms of TRP channel gating include DAG- and PIP2-dependent opening, as well as mechanosensory mechanisms (Liu and Montell, 2015). Similarly, the non-TRP channels that are opened in the newly discovered cascades proposed for M2 and M4 ipRGCs need to be identified, along with their gating mechanisms. In addition, the cascades initiated by the M3, M5 and M6 subtypes have not been examined, and this will be a necessary step in understanding their roles in behavior. Whether each of these new phototransduction cascades identified in mice are present in ipRGC subtypes across different species is an important area for future research, as are specific mechanisms within the G_q_/TRPC cascade that are present across species, including humans.

Thus, there is much to be reconciled on the question of ipRGC subtype phototransduction, and many other fascinating questions remain: What are the evolutionary origins of the newly uncovered melanopsin phototransduction cascades? Does the diversity of cascades identified in mouse persist in other species or are there additional pathways yet to be uncovered? What are the molecular, transcriptional underpinnings of this subtype diversity? Notably, research not directly related to the melanopsin phototransduction cascade itself will also be crucial to understanding melanopsin phototransduction. For example, work toward elucidating the crystal structure of melanopsin will be key to examining how precisely melanopsin binds to and activates G-proteins (and which G-proteins) structurally. Although homology modeling of mouse melanopsin to squid rhodopsin and the β-adrenergic receptor provides some insights into how G-protein(s) bind to melanopsin (Valdez-Lopez et al., 2020a), a crystal structure is required for definitive demonstration. The question of the bi-stability of melanopsin also may provide answers in regards to phototransduction; do each of the photostates influence structure to make melanopsin promiscuous?

For such an evolutionarily ancient and well-conserved visual pigment there is a striking amount of variety in its function. Much like the ipRGCs themselves, melanopsin phototransduction is more diverse than initially thought. New RNA sequencing datasets will likely allow for deeper insight into the diversity across and within ipRGC subtypes (Rheaume et al., 2018; Tran et al., 2019). There are multiple lines of evidence, for example, of diversity even within the M1 ipRGC subtype, but the molecular foundations of such diversity and any diversity in melanopsin phototransduction across M1 cells are unknown (Emanuel, et al., 2017; Lee, et al., 2019; Tran et al., 2019). Similarly, whether melanopsin phototransduction varies within other single ipRGC subtypes is an open question. Insights from other organisms clearly demonstrate the ability of melanopsin to be a promiscuous GPCR and support the novel research being done on melanopsin phototransduction in the mouse retina. As we gain more and more knowledge of the physical structure and properties of melanopsin, as well as an even more in-depth understanding of ipRGC subtypes in which it is expressed, we will gain a clearer picture of melanopsin phototransduction that will guide future work on non-image forming visual processes.

## Supplementary Material

Supplementary information
