# Supplementary material for: Melanopsin phototransduction: beyond canonical cascades
Source: J Exp Biol. 2021 Nov 29;224(23):jeb226522. doi: 10.1242/jeb.226522 (PMC8714064; doi:10.1242/jeb.226522)
Supplement: Supplementary information [file jexbio-224-226522-s1.pdf]

Journal of Experimental Biology: doi:10.1242/jeb.226522: Supplementary information

```
Mouse      1 MDSPSGPRVLSSLTQDPSFTTSPALQGIWNGTQN-VSVRAQLLSVSPTTSAHQAAAWVPF
Human      1 MNPPSGPRVPPSPTQEPSCMATPAPPSWWDSSQSSISSLGRLPSISPTAPGTWAAAWVPL
Chick X    1 MDLNMGTQ-PHSV-----------------------------------------------
Chick M    1 MDLP--PRAPTKMTVKD--------------------------------------VRGAF

Mouse     60 PTVDVPDHAHYTLGTVILLVGLTGMLGNLTVIYTFCRNRGLRTPANMFIINLAVSDFLMS
Human     61 PTVDVPDHAHYTLGTVILLVGLTGMLGNLTVIYTFCRSRSLRTPANMFIINLAVSDFLMS
Chick X   13 TKSEIPDHVLYTVGTCVLVIGSIGIIGNLLVLYAFYSNKKLRTPQNFFIMNLAVSDFLMS
Chick M   21 PTVDVPDHAHYTIGTVILIVGITGTLGNFLVIYAFCRSRTLQKPANIFIINLAVSDFLMS

Mouse    120 VTQAPVFFASSLYKKWLFGETGCEFYAFCGAVFGITSMITLTAIAMDRYLVITRPLATIG
Human    121 FTQAPVFFTSSLYKQWLFGETGCEFYAFCGALFGISSMITLTAIALDRYLVITRPLATFG
Chick X   73 ASQAPICFINSLHREWILGDIGCDLYAFCGALFGITSMMTLLAISVDRYLVITKPLRSIQ
Chick M   81 ITQSPVFFTNSLHKRWIFGEKGCELYAFCGALFGITSMITLMVIALDRYFVITKPLASVR

Mouse    180 RGSKRRTALVLLGVWLYALAWSLPPFFGWSAYVPEGLLTSCSWDYMTFTPQVRAYTMLLF
Human    181 VASKRRAAFVLLGVWLYALAWSLPPFFGWSAYVPEGLLTSCSWDYMSFTPAVRAYTMLLC
Chick X  133 WTSKKRTIQIIAAVWLYSLGWSVAPLLGWSSYVPEGLMISCTWDYVTYSPANRSYTMILC
Chick M  141 VMSKKKALIILVGVWLYSLAWSLPPFFGWSAYVPEGLLTSCSWDYMTFTPSVRAYTMLLF

Mouse    240 CFVFFLPLLIIIFCYIFIFRAIRETGRACEGCGESP--LRQRRQWQRLQSEWKMAKVALI
Human    241 CFVFFLPLLIIIYCYIFIFRAIRETGRALQTFGACKGNGESLWQRQRLQSECKMAKIMLL
Chick X  193 CCVFFIPLIIILHCYLFMFLAIRSTGRDVQKLGSCS---RKSFLSQSMKNEWKLAKIAFV
Chick M  201 CFVFFIPLIAIIYSYVFIFEAIKKANKSVQTFGCKHGNRELQKQYHRMKNEWKLAKIALI

Mouse    298 VILLFVLSWAPYSTVALVAFAGYSHILTPYMSSVPAVIAKASAIHNPIIYAITHPKYRVA
Human    301 VILLFVLSWAPYSAVALVAFAGYAHVLTPYMSSVPAVIAKASAIHNPIIYAITHPKYRVA
Chick X  250 VIIVYVLSWSPYACVTLIAWAGRGNTLTPYSKSVPAVIAKASAIYNPIIYAIIHPRYRKT
Chick M  261 VILLYVISWSPYSVVALVAFAGYSHVLTPFMNSVPAVIAKASAIHNPIIYAITHPKYRTA

Mouse    358 IAQHLPCLGVLLGVSGQRSHPSLSYRSTHRSTLSSQSSDLSWISGRKRQESLGSESEVGW
Human    361 IAQHLPCLGVLLGVSRRHSRPYPSYRSTHRSTLTSHTSNLSWISIRRRQESLGSESEVGW
Chick X  310 IHNAVPCLRFLIRISKNDLLRGSINESSFRTSLSSHQSLA-GRTK-NTCVSSVSTGEANW
Chick M  321 IATYVPCLGFLLRVSPKESRSFSSYPSSRRTTITSQSSETSGLQKGKRRLSSISDSESGC

Mouse    418 TDTETTAAWGAAQQASGQSFCSQNLED---GELKAS------------------------
Human    421 THMEAAAVWGAAQQANGRSLYGQGLED---LEAKAP------------------------
Chick X  368 SDVELDTVEPAHEKLQPRRSHSFSSSLRQKRDLLPDSYSCSEETEEKVSLSSSYLEKVLG
Chick M  381 TDTETDITSMISRPASSQVSYEMGEDTTQTSDLGGK--P-------K---VKSHDSGIFG

Mouse    451 -------SSPQVQRSKTPK--VPGPSTCRPMKGQGARPSSLRG--------DQKGRLAVC
Human    454 -------PRPQGHEAETPG------------KTKGLIP----------------------
Chick X  428 RSAFPSSPVALVTSSLRAASLPVGLNSSSASRGAGSDISQMKTEESHNNGGLDSIV----
Chick M  429 KAVVDADEIPMVEINDTEH---SATSTCKT--SEKCNVEEIQRSESLSGIGLREGESRRR

Mouse    494 TGLSECPHP------HTSQ-FPLAFLEDDVTLRHL-------------------------
Human    473 ---SQDPRM---------------------------------------------------
Chick X  484 --SNTVPQIIIIPTSETNL-FQEEPEEEETELFHFHDKKNNLLDLEGLSSSTEFLEAVEK
Chick M  484 TSASQIPSIIIT---YSNVQGVELPSGYSAGFLHPKNK----SHIQNKSSNS--------

Mouse        ---
Human        ---
Chick X  541 FLS
Chick M      ---
```

Transmembrane Region | Cytoplasmic Loop | Extracellular Loop | Amino Terminus | Carboxy Terminus

Fig. S1. Alignment: Sequence alignment of mouse, human, mammalian-like chicken (Chick M), and xenopus-like chicken (Chick X) melanopsins. An alignment of four melanopsin gene sequences (Uniprot, Appendix A) was created using T-Coffee and BOXSHADE (Notredame et al., 2000; Di Tommaso et al., 2011) Extracellular, intracellular, and transmembrane regions are denoted by overhead bars. The conserved lysine at mouse residue 291 is highlighted. High conservation is seen in the transmembrane regions, while less conservation is observed in both the amino (extracellular) and carboxy (intracellular) termini. Lower conservation in the third cytoplasmic (intracellular) loop as well suggests possible variations in G-protein specificity.

Journal of Experimental Biology • Supplementary information

Table S1. Uniprot Identifiers
Sequences for phylogenetic analysis and sequence alignment were retrieved from Uniprot. Accession numbers Q2KNE6 (Gallus gallus, Opn4m), A9QM68 (Gallus gallus, Opn4x), Q9QXZ9 (Mus musculus, Opn4), Q9UHM6 (Homo sapiens, Opn4), O57422 (Xenopus laevis Opn4b), Q2KNE5 (Danio rerio, Opn4a), F1QZ18 (Danio rerio, Opn4xa), E7FEB1 (Danio rerio, Opn4b), G8Z410 (Danio rerio, Opn4.1), G8Z413 (Danio rerio, Opn4xb), Q8R456 (Rattus norvegicus, Opn4), Q6XL69 (Rutilus rutilus, Opn4), Q4U4D2 (Podarcis siculus, Opn4), and Q4R1I4 (Branchiostoma belcheri, Opn4) were used.

**Accession Number**	**Accession Name**	**Organism**
Q2KNE6	Q2KNE6_CHICK (Opn4m)	Gallus gallus (Chicken)
A9QM68	A9QM68_CHICK (Opn4-1) (x)	Gallus gallus (Chicken)
Q9QXZ9	OPN4_MOUSE	Mus musculus (Mouse)
Q9UHM6	OPN4_HUMAN	Homo sapiens (Human)
O57422	OPN4B_XENLA	Xenopus laevis (African clawed frog)
Q2KNE5	OPN4A_DANRE (Opn4a)	Danio rerio (Zebrafish) (Brachydanio rerio)
F1QZ18	F1QZ18_DANRE (Opn4xa)	Danio rerio (Zebrafish) (Brachydanio rerio)
E7FEB1	E7FEB1_DANRE (Opn4b)	Danio rerio (Zebrafish) (Brachydanio rerio)
G8Z410	G8Z410_DANRE (Opn4.1)	Danio rerio (Zebrafish) (Brachydanio rerio)
G8Z413	G8Z413_DANRE (Opn4xb)	Danio rerio (Zebrafish) (Brachydanio rerio)
Q8R456	OPN4_RAT	Rattus norvegicus (Rat)
Q6XL69	OPN4_RUTRU	Rutilus rutilus (Roach)
Q4U4D2	OPN4_PODSI	Podarcis siculus (Italian wall lizard)
Q4R1I4	OPN4_BRABE	Branchiostoma belcheri (Amphioxus)

References

Di Tommaso, P., Moretti, S., Xenarios, I., Orobitg, M., Montanyola, A., Chang, J. M., Taly, J. F. and Notredame, C. (2011). T-Coffee: a web server for the multiple sequence alignment of protein and RNA sequences using structural information and homology extension. *Nucleic Acids Res.* **39**, W13-W17. doi:10.1093/nar/gkr245

Notredame, C., Higgins, D. G. and Heringa, J. (2000). T-Coffee: a novel method for fast and accurate multiple sequence alignment. *J. Mol. Biol.* **302**, 205-217. doi:10.1006/jmbi.2000.4042
